# Everybody's Page

**Published:** 1888-12-15

**Authors:** 


### Everybody's Page.

There are five hospitals for leprosy in Norway, three of which are in Bergen.

Madness has been known to result from prolonged exposure to cold, especially if this be accompanied by hunger.

The manifestations of lunacy are generally more dangerous in the male sex. It is very rare for a woman to suffer from homicidal mania, and the statistics of suicide show that the majority of those who kill themselves are men.

Dr. J. Roussel is of opinion that gold is the best cure for anæmia among the blonde races of northern Europe. This sounds very pretty, but the general opinion is that iron is of more practical value.

It is generally known in a vague way that old men lose a little in height. Quetelet, who studied the matter closely, calculated that the shortening began at fifty, and that if a man lived till he was ninety he would be an inch and a-half shorter than he was in his prime.

A child first begins to smile when about six or seven weeks old. This is its first voluntary expression of pleasure, and it usually springs from seeing its mother's face. Six weeks later it will begin to smile with amusement at something of the nature of play, and after that time the capacity for humour develops rapidly.

One of the latest articles of clothing against which the preachers of hygiene have ranged themselves is the stiffly-starched shirt-front. What is the good, asks the physiological satirist, of gluing two or three folds of linen together in this way? It not only makes the clothing less useful, but it a waste of valuable food.

There are in India 213 separate species of snakes, but only 33 of these are poisonous. A line of carbolic acid powder across the doorway is effectual in preventing a snake from entering a room, as the reptiles have a great aversion to this useful antiseptic. Unfortunately, it is equally effectual in preventing the egress of a snake that happens to be inside.

The following announcement appeared some time ago in a Glasgow paper: "James Hodge continues to sell burying crapes ready made; and his wife's niece dresses dead corpses at as cheap a rate as was formerly done by her aunt, having not only been educated by her, but perfected in Edinburgh, from whence she has lately arrived, with all the newest and best fashions for the dead."

It would be interesting, if somewhat sad, to discover how many children whose parents are comparatively rich are starved to death, not from under-feeding, but from unsuitable food. In some cases it is all meat and no milk, in others all milk and no meat; and in either case the child lacks some of the constituents of flesh, blood, and bone. Many parents seem to think that sameness in food is identical with simplicity, and pride themselves on the virtue of a course of action which is nothing less than murderous.

The Countess of Southdown, as is known to all readers of "Vanity Fair," used to dose her protégés and sycophants and their families with Daffy's Elixir, Rodgers' Pills, Podgers' Pills, and other medicaments, at her noble pleasure, and none of the recipients ventured on a murmur. But truth goes beyond fiction, and in the last century, according to Lord Chesterfield, those who wanted to curry favour with a patron cursed with a belief in phlebotomy used to shed their blood for him, or at least let it be shed by his lordship's surgeon to the extent of ten or twelve ounces, and pretend they liked it.

There is a proverb to the effect that Providence looks after children and drunken men—that is, in preventing them from sustaining serious damage from their frequent falls. The softness and elasticity of the bones of the more innocent sufferers may in part account for their immunity from accident, but their safety in part depends, as does that of the inebriates, on their making no effort to avert the catastrophe. Both let themselves fall without struggling to save themselves, and so escape those wrenches and sprains which are often the worst results of a fall.

Long hours of work are often as little to the employer's interest as they are to those employed. No one can do as good work at the end of the day as at the beginning, and the more the working hours are prolonged the greater will be the deterioration of the work, and as the time for recuperation must inevitably be shortened the deterioration will very probably become continuous. Sir Thomas, now Lord Brassey, in his book on "Work and Wages," speaks of a certain gang of navvies who worked on the Great Northern Railway. They did more work in a day than any other gang on the line, and always left off work an hour and a-half earlier than any other.

The member of our staff who recently protested so earnestly against the use of birds as trimmings for hats, should be thankful that he did not live in the fifteenth century. In the year 1434 Thomas Conecte, a Carmelite monk, was burnt at the stake. His chief offence was the vehemence of his sermons against the enormous head-dresses worn by the ladies of his day. They were so high and so broad that a fashionable woman could not get through an ordinary doorway without stooping and going sideways. And because Conecte did not admire this fashion they burned him. What doom, we wonder, would be meted out to our *confrère* by a jury of milliners?

One of the rules most needful to be impressed, and most difficult to impress, on the untrained or amateur nurse, is that in typhoid fever, diphtheria, scarlet fever, measles, and whooping-cough it is necessary to pay the greatest attention to the patient after the disease is apparently cured. The sequelæ of these ailments are often the most dangerous parts of them. It is often when the patient's friends think and hint that the doctor need not come quite so often that the real danger begins, for then the seeming convalescent will get wrong food, and receive too many visits, and generally be treated with a mixture of coddling and neglect that often terminates fatally.

A pretender to the uncomfortable honour of being blind of the left eye, who had pecuniary reasons for claiming this misfortune, was cleverly found out not long ago. All the old tests, cures, and so forth were tried in vain, when a shrewd expert, who recalled M. Chevreul's laws as to complementary colours, invented a new one. He traced a few words in green on a black screen. Then he placed on the patient's nose a pair of spectacles, which had a red glass for the right eye and a white one for the left, and asked him to read the words he had written. Without distrust or hesitation, the man read out the words. "Are you sure it is with the right eye you have been reading?" asked the doctor. "Certainly," answered the patient; "I can't see with the left." "But," returned the doctor, "that is not possible; for as red is the complementary colour of green, the red glass before the right eye effaces them for that eye, and confounds them with the black of the screen. It is with the left eye, through the white glass that you have seen the words." And the patient went away a sadder man, though his vision was pronounced perfect.

